# New media in family sustainability: mechanisms for building social resilience through awareness programs

**DOI:** 10.3389/fsoc.2026.1761970

**Published:** 2026-03-18

**Authors:** Alaa Makki Abdulhadi Akkof, Rahima Aissani, Amirah Saad AlZahrani, Ibrahim Ukka, Islam Habis Mohammad Hatamleh

**Affiliations:** 1 Department of Mass Communication, College of Communication, University of Sharjah, Sharjah, United Arab Emirates; 2 College of Communication and Media, Al Ain University, Al Ain, United Arab Emirates; 3 Department of Curriculum and Instruction, College of Education and Human Development, University of Bisha, Bisha, Saudi Arabia; 4 Department of Communication and Digital Media, Najah National University, Nablus, Palestine; 5 Department of Media and Communication Technology, Faculty of Media, Jadara University, Irbid, Jordan

**Keywords:** family sustainability, new media, post COVID 19, resilience, society

## Abstract

New media are playing an increasing role in promoting family sustainability. They are no longer merely entertainment platforms, but rather awareness-raising and educational tools that contribute to building social resilience within society. Through digital awareness programs, families can acquire skills to adapt to economic, cultural, and technological challenges and prevent potential crises. This study examines the influence of new media on promoting family sustainability and enhancing social resilience via awareness initiatives, specifically targeting Jordanian families. In-depth semi-structured interviews were done with 12 participants selected purposively and through snow ball sampling technique from various family configurations in Jordan using qualitative approaches. The interviews examined how new media platforms, including social media networks, facilitated family connectedness, disseminated vital information, and bolstered emotional well-being during difficult periods. Thematic analysis identified essential mechanisms via which new media promoted resilience-building, such as the distribution of health information, participation in community support programs, and the establishment of virtual social networks. The findings also underscored the difficulties related to misinformation and digital disparity. Research indicates that strategically utilized digital media can empower families, enhance collective resilience, and offer significant emotional and social support, particularly in times of crisis. This study enhances the existing research on digital tools for family sustainability, offering perspectives on the efficacy of new media as a resilience-building mechanism in Jordanian communities.

## 1 Introduction

The durability of family groups has emerged as a crucial field of research, especially with social resilience in an increasingly linked society (Davydov et al., 2010). Social resilience denotes the ability of people and communities to adapt to and recuperate from adversity, hence safeguarding the well-being and continuation of familial structures throughout crises (Yun and Kim, 2023). The emergence of new media has created novel opportunities for cultivating resilience, including platforms for knowledge distribution, emotional assistance, and community involvement (Heponiemi et al., 2023). The COVID-19 pandemic highlighted the crucial importance of internet platforms in preserving familial unity and support networks (Habas and Abu Alasal, 2025; Khasawneh et al., 2025). Abdulai et al. (2021) studies demonstrate that social media and online communication platforms were vital in overcoming the isolation caused by physical separation measures, allowing families to maintain connections and exchange essential information (Burke et al., 2010). These platforms enabled the dissemination of health recommendations, instructional materials, and emotional support; therefore, they enhanced the psychological well-being of family members. Gao et al. (2020) examines the convergence of new media with family dynamics and cultural norms. Cultural norms in Jordan profoundly anchor family life, prioritizing intimate connections, familial responsibilities, and communal interdependence (Aljedayah et al., 2024). The nation gets more digitally interconnected, there is an escalating need to comprehend the interaction between conventional values and the novel chances and problems presented by digital platforms (Habes et al., 2025). It is essential to include digital literacy programs and family resilience strategies to assist families in identifying reputable sources and circumventing disinformation, especially during periods of uncertainty (Magis, 2010).

In Jordan, a nation defined by strong family connections and community relationships, the use of new media has been notably impactful. Research has shown how Jordanian families used internet platforms to address the problems presented by the pandemic, including homeschooling, remote employment, and obtaining healthcare information (Mohammad et al., 2024).

This paper dwell on how the new media has become more than entertainment that is fundamental in ensuring awareness creation and education that enhances family sustainability and social resiliency in families of Jordan. These digital platforms specifically, messaging apps (e.g., IMO and Messenger), and digital forums that support the sharing of information and emotional support. Notwithstanding the extensive use of these technologies, there is a deficiency in studies investigating their particular effects on family sustainability and resilience in the Jordanian setting. This research seeks to investigate the processes by which new media awareness initiatives enhance family sustainability and societal resilience in Jordan. This study employs qualitative interviews with 12 Jordanian families to ascertain how digital platforms promote information dissemination, emotional support, and community involvement, ultimately bolstering family resilience.

Research questions:

1. In what ways do new media platforms boost family resilience during crises in Jordan?
2. What strategies in new media awareness initiatives promote knowledge distribution and enhance social support networks among families in Jordan
3. In what ways do new media platforms influence the mental health and emotional fortitude of families in Jordan during times of adversity?

## 2 Literature review: new media in family sustainability and resilience

### 2.1 Family sustainability and social resilience at global level

Family sustainability denotes a family’s capacity to preserve its fundamental functions–emotional, economic, and social while adjusting to crises, economic adversities, or societal changes (Kimhi and Shomai, 2004). Social resilience, especially regarding family dynamics, refers to the capacity to withstand and adjust to disruptions such as natural catastrophes, pandemics, or economic instability, while maintaining core family values and support structures. Norris et al. (2008) explained Families, as fundamental components of society, are essential to the social structure. Their resilience is often challenged during crises, necessitating an understanding of how they manage adversity while preserving their fundamental operations (Sippel et al., 2015).

The significance of new media in family sustainability has grown, particularly due to the emergence of digital technologies that enhance communication, connection, and social support. Happer and Philo (2013) contemporary media channels, including social media, mobile apps, and digital forums, provide unparalleled access to information, facilitating familial connectivity, resource sharing, and psychological support during difficult periods (Jane et al., 2018). The COVID-19 pandemic heightened the dependence on new media, demonstrating its ability to enhance family resilience. Goodyear et al. (2021) told that, nonetheless, despite its promise, the investigation into the specific processes through which new media fosters family sustainability is still little examined. New media channels have transformed the manner in which families obtain and share essential information, especially during crises (Kudchadkar and Carroll, 2020). Throughout the COVID-19 pandemic, governments and health organizations, including Jordan, used digital channels to provide timely information on health protocols, vaccination initiatives, and emergency response strategies. The convergence of new media and mental health has attracted much scrutiny in recent years (Croghan et al., 2021). The impact of social media on mental health is multifaceted; it may provide emotional support; however, it may also exacerbate stress, anxiety, and depression when used excessively or carelessly (Özmete and Pak, 2020). Research indicates that during crises, excessive social media use may intensify emotions of isolation, worry, and panic, particularly when families get unpleasant news (Holmes et al., 2020).

### 2.2 Social resilience at the regional/local level

Arab families used platforms such as Messenger, Facebook, and IMO to acquire information on health measures, online education, and economic assistance programs (Jiang et al., 2022). The rapidity and availability of these platforms enabled families to remain informed, adapt swiftly to changing circumstances, and make choices that safeguarded their safety and welfare (Al-Dmour et al., 2022). The effectiveness of new media in disseminating information relies on several elements, such as digital literacy, technological access, and the reliability of information sources. Ahmed et al. (2023) assert that the abundance of disinformation on social media platforms presents a substantial obstacle to efficient information distribution.

To enhance family sustainability and resilience, new media platforms must provide families with the necessary skills for responsible and successful use. Gadjanova et al. (2022) explained that media literacy programs are crucial for assisting families in navigating digital environments. These programs educate persons in the use of technology and the critical evaluation of online content (Houston et al., 2017; Islam Habis Mohammad Hatamleh, 2023). In Jordan, media literacy efforts have proliferated, especially via partnerships between governmental entities and non-governmental organizations. These initiatives emphasize digital literacy among parents and children, empowering them to use new media for resource access, information sharing, and crisis assistance. Alghizzawi et al. (2018) alongside media literacy, the creation of collaborative platforms tailored for family assistance has shown significant value. These platforms enable families to collaborate on problem-solving, exchange information, and provide emotional support (Ferreira and Pantidi, 2017). These platforms provide a viable method for fostering enduring family sustainability by cultivating a feeling of community and collaborative activity, even with physical separation. Kimhi and Shomai (2004) by cultivating online support networks, these platforms guarantee that families obtain essential services and are more adept at managing emergencies. Masten (2018), although digital media offers several opportunities to bolster family resilience, it also poses some obstacles. A significant barrier is the digital divide, which restricts the capacity of some families, especially those in rural or economically disadvantaged regions, to effectively use digital resources (Jiang et al., 2022). Families without dependable internet connections or cellphones may find it challenging to participate in new media initiatives, so diminishing their capacity for resilience-building (Cuervo et al., 2017). Mitigating this digital gap is essential for enabling new media to serve as a mechanism for family sustainability within various socio-economic strata (Zhang and Shay, 2019). The excessive use of digital platforms might result in digital overload, adversely affecting mental well-being (Burke et al., 2010). Continuous exposure to news, particularly during emergencies, may exacerbate stress and anxiety (Karmegam et al., 2019). It is essential to equilibrate the use of new media with offline pursuits, including in-person conversation, physical exercise, and relaxation, which enhance general well-being (Teague et al., 2022). New media platforms have revolutionized familial interactions and resource accessibility, significantly contributing to the enhancement of family resilience amid crises. According to Happer and Philo (2013) these platforms enable information distribution, enhance social support networks, and provide channels for emotional assistance, thereby promoting better mental health. Janků et al. (2022) to enhance the efficacy of new media in advancing family sustainability, we must confront obstacles such as the digital gap, disinformation, and digital saturation. Heponiemi et al. (2023) emphasize the significance of media literacy initiatives and cooperative digital platforms in bolstering family resilience and supporting families during challenging periods.

Social support is essential in promoting family resilience during crises (Croghan et al., 2021). Despite the fact that this research paper uses the definition of social support as the help that people obtain through new-media-induced interactions, one should be aware of the fact that the social support construct is a complex one. Researchers have determined three main dimensions, including emotional support (indications of empathy, care, and reassurance), instrumental support (concrete support and practical assistance), and informational support delivery of guidance, advice, or knowledge (Sippel et al., 2015; Özmete and Pak, 2020). A combination of these forms leads to resilience-building in families, especially through digital platforms where interpersonal communication takes place through mediating channels. Emotional support provides psychological stability and connectedness, instrumental support alleviates practical burden in crisis and informational support improves decision making and coping efficacy (Duong et al., 2025). Thus, regarding this study, social support can be treated as a multidimensional process that involves emotional, instrumental and informational aspects, that can be provided through new media platforms to enhance family resilience and sustainability during crises (Al-Dmour et al., 2022). New media channels are crucial for enhancing familial bonds, both inside homes and with extended family and the wider community. Throughout the pandamic, several families in Jordan and the Arab world used social media to maintain connections with family, friends, and neighbors (Jiang et al., 2022). These forums facilitated families in sharing experiences, exchanging coping solutions, and providing emotional support, therefore mitigating feelings of isolation and worry.

The sense of collectivism and strong family ties in Jordanian families provide a substantial level of emotional support and make the family more resilient in times of crisis. These ties are, however, also known to restrict information exchange and social mobilization because they emphasize mainly on internal support (Semmer et al., 2008). Such an introspective can inhibit availability of more resources or external knowledge and this could impair the coping mechanisms (Sippel et al., 2015). Therefore, the discussion on the positive and negative sides of such networks should be taken critically in order to comprehend their full effects on family resilience.

Islam Habis Mohammad Hatamleh (2023) discovered that Jordanian families, particularly in rural regions, gained advantages from digital support networks offering psychological assistance and direction (Sarwar et al., 2023a). During the pandemic, online support groups, mental health forums, and community chat groups emerged as essential tools for families seeking solidarity and guidance. The capacity to participate in these virtual communities enabled families to sustain robust social connections, a crucial element of resilience development (Heath et al., 2020).

In Jordan, where familial unity and support are vital for managing hardship, new media channels have played a crucial role in promoting mental well-being throughout the pandemic Aljedayah et al. (2024) indicates that Arab families who use social media to share experiences and access mental health services have improved coping mechanisms and emotional resilience (Moitra et al., 2018). Platforms like Facebook and Instagram enabled consumers to engage with mental health specialists, utilize online counseling services, and participate in virtual courses focused on emotional well-being (Ferreira and Pantidi, 2017). The advantages of new media are not always applicable. Sarwar et al. (2023a) emphasize that digital platforms may exacerbate mental health difficulties, especially when misinformation proliferates or when consumers are inundated by incessant news cycles (Elmer et al., 2020). The dual capacity of social media to both enhance and detrimentally affect mental health necessitates greater investigation, particularly with family interactions in times of crisis.

The two theoretical frameworks that guide this study are the Resilience Theory and the Social Support Theory. The concept of resilience theory focuses on how individuals or families can adapt and survive despite challenges. It emphazises the importance of internal and external resources, including family processes and social networks in crisis overcoming (Masten, 2018). Family resilience in this research is taken to mean the ability of the family members to sustain their psychological well-being, emotional stability and functional efficiency during stressful situations and is largely mediated through digital means. The Social Support Theory as expressed by Cohen and Wills (1985) assumes that support offered by the social network, whether in the emotional, informational or instrumental form can greatly contribute to the capacity of an individual to deal with stress and thus be resilient. The collectivist aspect of family networks that are prevalent in Jordanian families is core in offering emotional support, which in its turn ensures the resilience of the family. The framework represents the multidimensional contribution of social support to the coping processes and decision-making in a family setting, especially during crisis periods. By exploring the multidimensional nature of social support within the context of Jordanian families, this study aims to illuminate the dynamics that enhance or hinder family resilience, particularly when mediated through digital platforms, there is a significant gap in research regarding how these support systems operate specifically within the Jordanian context, especially when digital tools play a central role.

## 3 Methodology

### 3.1 Research design

This qualitative study examined how new media promotes family sustainability and societal resilience via awareness campaigns in Jordanian households (Ali et al., 2021; Ullah et al., 2024; Al-Shakhanbeh and Habes, 2022). The qualitative approach is best for understanding the many contextual factors that impact family dynamics and resilience, which quantitative methods may miss (Yadav, 2022). A qualitative study illuminates participants’ lives, beliefs, and how modern media affects family resilience. Miles and Huberman (1984) said that semi-structured interviews were used to collect data since the study was exploratory Ethical approval was obtained from the Research Ethics Council of Jadara University in Jordan, number R.5.2.2025. This technique ensures the inclusion of important research topics in interviews while allowing for flexible, extensive responses. The sampling method was purposive; therefore, participants who had certain experience regarding family in digital contexts were targeted. Also, snowball sampling was used to enlarge the sample because initial respondents referred others (Patton, 2002; Safeer et al., 2023). This was a combination that guaranteed a wide but pertinent sample (Alhanatleh et al., 2023; Habes et al., 2022). All the participants actively informed consent and were thoroughly informed of the objective of the study, the voluntary nature of the data gathering and the privacy. To make sure that there is sample diversity and to facilitate the analysis of data, the survey included sociodemographic information, such as age, gender, family set-up, and level of education (Cresswell, 2014). The data gathaered in a period of 4 weeks, Semi-structured interviews enable researchers to explore family resilience, new media, and awareness initiatives’ resilience-building effects (Hammarberg et al., 2016).

### 3.2 Sample

Strong cultural and societal standards influence family relations in Jordan, the study’s location. Jordan, like other Arab nations, struggled with distant schooling, economic hardship, and social isolation during the COVID-19 pandemic. It offers a framework for studying how new media technologies might strengthen families. Interviewees were chosen using purposive sampling. The researcher picked this non-random selection strategy because it enables them to choose examples of the issue under investigation, such as Jordanian families that have used new media amid crises. Our sample included 12 Jordanian families from various socioeconomic origins, urban and rural settings, and family types (nuclear, extended, and single-parent). In qualitative studies the data saturation principle often justifies a sample of 12, where data collection does not indicate much further information. In case of specialized reports such as new media mechanisms in family sustainability, this number corresponds to empirical data that saturation usually falls in a limited range of 9–17 interviews (Hennink and Kaiser, 2022). The sample diversity guarantees that the results represent diverse family experiences and digital participation. We recruited participants using social media, community groups, and personal networks (Megdadi et al., 2023). To reach diverse demographics, recruitment communications were in Arabic and English. The research family has to satisfy these criteria: Needed to use Facebook, messenger, imo, and other social media apps during COVID-19 and post covid times. they must have utilized online assistance or engaged in digital awareness exercises. Must accept a semi-structured interview.

### 3.3 Data collection

Data was collected mostly via semi-structured interviews. The flexible interviews enabled the researcher to study family resilience and new media participation in detail while following a defined interview process to maintain consistency (Duggleby et al., 2020). The literature review and research questions informed the interview approach. For participants to openly communicate their experiences and ideas, the procedure contained open-ended questions in the interview Guide (Sarwar et al., 2023b; Ziani et al., 2021). The queries were as follows.

- What new media did your family utilize during the COVID-19 pandemic to remain connected and get critical information?
- What digital awareness initiatives or online tools did your family employ during the pandemic?
- How did these digital tools improve your family’s emotional and practical pandemic resilience?
- Did modern media aid or hurt your family’s mental health amid crises?
- Did your family have any trouble utilizing modern media for crisis help and information?

Participants preferred and were available for in-person interviews. Each interview lasted 45–60 min. The researcher established rapport to make participants feel comfortable expressing their stories. Participants were interviewed in Arabic or English, depending on their choice. Audio-recorded and verbatim interviews were conducted with permission. Arabic transcriptions were translated into English. The researcher assigned pseudonyms and securely stored data to protect participant privacy. Semi-structured interview data was analyzed using thematic analysis, a popular qualitative data analysis approach (Braun and Clarke, 2006). Data themes may be identified, analyzed, and reported using thematic analysis. This technique helps explain how participants interpret their experiences and how topics, such as the significance of new media in family resilience, emerge during interviews.

The analysis followed Braun and Clarke (2006) six steps.

1. *Data familiarization*: I typed the interviews as they were and, after that carefully read the transcripts several times to get familiar with the data. This exercise allowed me to know the general context of the responses of the participants, and I proceeded with the coding.
2. *Initial coding*: At this stage, I was able to identify and mark the useful bits of data that applied to the research questions. I have then coded these segments with initial codes. As an example, any reference involving emotional support through social media was classified as emotional support and social media use.
3. *Themes searching*: Once the initial coding was done, I combined similar codes to create bigger themes. As an illustration, emotional support, virtual communication, and social connection codes were combined as the theme of Family Resilience via Social Media suring crisis.
4. *Reviewing the themes*: I have been able to revise the themes by going back to the coded data and making sure they capture the experience of the participants. This was also a step of refining the themes to ensure that they were unique and in line with the questions of the study. I removed all redundant or overlapping themes.
5. *Defining and naming themes*: After refining the themes, I named them in a manner that best defined the data. As an illustration, the theme of Accessing Resources was determined to reflect the use of new media by the families to access information on health as well as financial aid, among other services.
6. *The report production*: At last, I composed the results, using direct quotes of the participants to substantiate the themes. I also drew comparisons between these themes and already existing literature on the new media and family resilience to bring out the specific findings as well as the general implications.

## 4 Analysis

**Figure 1:**
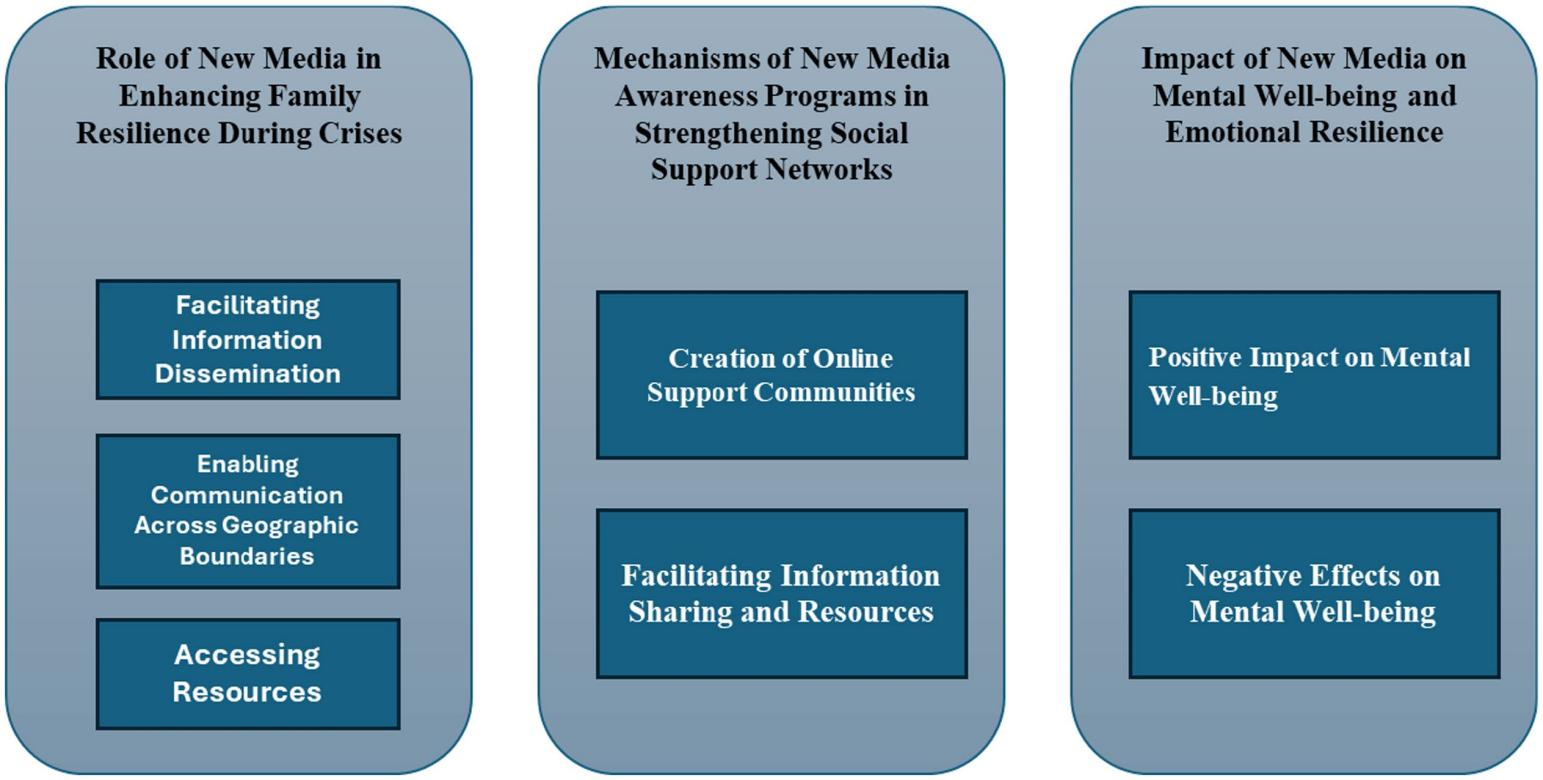
Primary themes and subthemes.

The following analysis uses semi-structured interviews with 12 Jordanian families to examine how new media platforms help families cope with emergencies like the COVID-19 outbreak. The core research questions guide the analysis, which highlights interview topics (Figure 1).

### 4.1 Role of new media in enhancing family resilience during crises

The initial research question of this study is to assess the role of new media platforms in bolstering family resilience during crises in Jordan. The findings indicated that new media platforms significantly contributed to bolstering family resilience by allowing information exchange, permitting communication across geographical boundaries, and assisting families in accessing resources.

#### 4.1.1 Facilitating information dissemination

A key function of new media in bolstering family resilience is its capacity to swiftly disseminate essential information. Croghan et al. (2021) families indicated a significant dependence on platforms like Facebook, Messenger, and imo for information pertaining to health procedures, educational resources, and economic support during the COVID-19 pandemic.

Participant 1 stated: “*During the lockdown, we utilized imo to maintain communication with relatives and to receive updates regarding government health guidelines. It was the most expedient method to get information on all occurrences in our vicinity*.”

Participant 7 reiterated this view, asserting: “*We joined a imo group for Jordanians during the pandemic.” We received consistent information about food delivery services, medical aid, and governmental pronouncements. It significantly aided in ensuring our family’s safety and awareness*.”

This illustrates Participant 1 also says that IMO was the quickest method of communicating with their relatives as well as being informed about the health requirements in the government. It made them have fast access to information about what was going on around them. Participant 7 adds that they joined a special IMO group among the Jordanians in the midst of the pandemic. They were constantly updated regarding food delivery, medical assistance, and the government in this group. This aided them in preserving their family safety and knowledge.

#### 4.1.2 Enabling communication across geographic boundaries

A significant contribution of new media to family resilience was its capacity to sustain contact among family members, even those separated by lockdowns or travel restrictions. Families in Jordan, especially those with extended relatives, used new media channels to remain connected, provide emotional support, and sustain familial cohesiveness throughout crises.

Participant 3 stated: “*My parents reside in a different city, and we were unable to visit them due to the lockdown. However, we used imo almost every day. It fostered a sense of closeness and provided the necessary emotional support*.”

Participant 5 said, “*When the schools closed, my children were engaged in online classes. I was unable to be with them constantly; nevertheless, we were still able to discuss their homework and academic assignments via Messenger and IMO*.”

The results of Participant 3 discuss the way in which his/her parents reside in another city, and due to the lockdown, they were not able to see them. However, they communicated daily using IMO, and this made them feel close and emotionally supported even when the distance existed. Participant 5 tells how their children had attended online classes during the pandemic. Although they are unable to spend the majority of their time together, they could still assist with homework and academic tasks and communicate through Messenger and IMO. In both instances, the participants are sharing how IMO and Messenger benefited them to be close to their family and support them in both emotional and academic life in the course of the lockdown.

#### 4.1.3 Accessing resources

Another crucial function of new media was to provide access to resources, including mental health treatments, educational materials, and economic assistance. Numerous participants indicated using social media platforms and mobile apps to get information on financial assistance programs, health resources, and counseling services.

Participant 4 emphasized: “*I utilized a mental health application suggested by a friend on IMO. It assisted me in maintaining composure and directed me through breathing techniques when I felt inundated by the circumstances*.”

The capacity to access online resources was essential for families, particularly when conventional support methods, such as in-person gatherings, were inaccessible due to the pandemic.

### 4.2 Mechanisms of new media awareness programs in strengthening social support networks

The second study question was to investigate the processes within new media awareness initiatives that promote knowledge distribution and enhance social support networks among families in Jordan. The findings indicated that digital awareness initiatives fostered supportive settings, enabling families to exchange advice, share resources, and connect with others facing analogous issues.

#### 4.2.1 Establishment of virtual support communities

A fundamental technique for enhancing social support was the establishment of online communities, where people could exchange experiences, provide advice, and furnish emotional support. Numerous families indicated their participation in Facebook groups and IMO groups inside communities and online forums particularly established to assist families throughout the pandemic. These platforms enabled families to connect with others in like circumstances, exchange coping skills, and participate in collective resilience enhancement.

Participant 6 said, “*I joined an online group for parents in Jordan. The community was very supportive, providing a forum for me to express my concerns for my children’s mental health, while others recounted their experiences and offered advice on homeschooling during the lockdown*.”

Participant 8 also stated: “*There was an IMO group for families in our neighborhood.” We exchanged advice on maintaining health and strategies to assist our children in managing isolation, and we also shared food supplies with families in need*.”

This illustrates how new media enabled the establishment of supportive online networks that offered essential resources, both practical and emotional, at a period of communal distress.

#### 4.2.2 Promoting information exchange and resource allocation

In addition to emotional support, digital awareness initiatives also enabled the dissemination of practical information and tools. Numerous participants observed that new media platforms facilitated access to an abundance of information on pandemic coping strategies, including educational resources for children, financial assistance programs, and government laws.

Participant 9 stated: “*We utilized the messaging application imo to disseminate diverse resources, including complimentary online courses, information regarding government assistance programs, and health recommendations. It was an excellent method of providing mutual assistance during challenging periods*.”

Participant 2 said, “*I discovered a mental health hotline via a Facebook page. In its absence, I would have been uncertain about how to manage my anxiety throughout the lockdown*.”

These replies underscore the significance of new media in enabling the dissemination of essential information, allowing families to assist one another via practical tools and assistance. According to Participant 9, they utilized IMO, which is a messaging application, to exchange numerous beneficial materials such as free online courses, details on government aid initiatives, and health tips. It was an excellent method of supporting one another in bad times. Participant 2 says that he found a mental health hotline on a Facebook page. In the absence of this resource, they would not have known how to deal with the anxiety imposed by the lockdown. The two interviewees are conversing about their use of social media and messaging applications to find the necessary resources and support in the pandemic.

### 4.3 Influence of new media on psychological well-being and emotional resilience

The third study question examined the influence of new media platforms on the mental health and emotional resilience of families in Jordan during periods of distress. The results indicate that while new media might improve mental well-being by offering emotional support and fostering connections, it may also yield adverse effects if excessively used or improperly employed.

#### 4.3.1 Beneficial effects on mental well-being

For several families, new media channels provided a crucial method for managing the emotional difficulties presented by the pandemic Participants said that using digital technologies to maintain connections with loved ones, participate in online support groups, and access mental health services positively influenced their emotional resilience.

Participant 11 said, “*At the onset of the pandemic, I experienced significant stress and isolation. However, communicating with my friends and family on Facebook, in my opinion, improved my mood. I discovered many online therapy groups that assisted me in managing my anxiety*.”

Participant 12 affirmed, stating: “*Social media enabled me to maintain contact with my family, and I discovered several mental health groups that assisted me in coping*. It *was preferable to just consuming news online, which was causing me considerable* worry.”

These assertions indicate that new media platforms were crucial in fostering emotional resilience, facilitating connections, and granting access to mental health resources. Participant 11 tells that at the beginning of the pandemic, he or she felt stressed and isolated. Yet, through constant communication with their friends, and family on face book, they felt better and found themselves in a better mood. They also discovered the online therapy groups which made them cope with their anxiety. Participant 12 concurs with this, indicating that the social media enabled them to keep in touch with their family. They also identified mental health groups over the internet who helped them to deal with their feelings. They believed that their participation in such groups was far more superior to simply reading news on the internet, which was causing them to become increasingly anxious.

Both participants emphazise the fact that social media enabled them to remain connected, seek emotional support, and manage the pandemic stress.

#### 4.3.2 Adverse impacts on psychological well-being

Notwithstanding the advantages, new media also posed problems to mental well-being. Numerous participants observed that the overuse of social media, exposure to troubling news, and misinformation exacerbated worry and stress throughout the pandemic.

Participant 3 states: “*Occasionally, the news on Facebook induced anxiety in me. I had to stop browsing temporarily due to feeling inundated by the plethora of nasty postings*.”

Participant 10 said, “*There was an abundance of misinformation circulating on social media, which caused me to concern myself with issues that were unwarranted. Determining what to believe was challenging*.”

These results demonstrate the potential adverse consequences of new media, especially with disinformation and excessive exposure to unpleasant material. The capacity to regulate and oversee digital media intake is essential for maintaining mental health. Participant 3 states that they occasionally felt anxious because of the news they received in Facebook. Participant 10 discusses that social media was full of false or misleading information that made them need to take a break in browsing the content. It is causing them to be concerned about what was not even real, and it was difficult to discern what information they could rely on in either situation due to the massive deluge of misleading or even negative information.

## 5 Discussion

Our study sample (*n* = 12) yielded numerous critical conclusions about experiences of social media-related resilience, especially during crises like the COVID-19 pandemic, based on comprehensive interviews and a qualitatively rich thematic analysis. This study’s results provide insights into how social media platforms function as essential instruments for cultivating resilience at both individual and group levels. This analysis addresses resilience-building initiatives, resource-sharing, and prosocial behavior, emphasizing their manifestation in densely populated metropolitan environments like Jordan. Our results demonstrate that social media significantly contributed to the enhancement of both individual and community resilience. The results of this research align with the findings of Mohammad et al. (2024), who propose that social media may be used as a medium for cultivating resilience in many manifestations. Our study indicated that during periods of confinement, especially in densely populated metropolitan areas such as Jordan, inhabitants used social media to preserve a feeling of normality and purpose. Social media sites offered users the option to explore interests, participate in creative pursuits, and sustain mental well-being throughout the lockdown. Numerous participants indicated using platforms like Instagram and Facebook to establish devoted profiles for their creative pursuits, including painting, cooking, and music. For example, one participant shared, he started an Instagram page for his art, which kept him motivated and gave him a sense of achievement during the lockdown. This correlates with the positive psychology paradigm, where pursuing personal interests and retaining a sense of purpose have been found to promote resilience during situations of crisis (Leykin et al., 2013). A feeling of purpose mitigates negative emotions by fostering optimism and enhancing emotional well-being. Participating in creative endeavors and disseminating these achievements on social media cultivates an optimistic perspective, which, as prior research indicates, correlates with improved mental health outcomes (Jin, 2009).

Moreover, our research corroborates the claim of (Sippel et al., 2015) that social media platforms facilitate the interchange of health and wellness ideas among community members. Participants said that they were motivated by others to partake in physical activities, including yoga and home workouts, often disseminated via Instagram postings and YouTube videos. This underscores social media’s function in improving mental well-being and fostering healthy habits, hence enhancing resilience in times of stress (Barry et al., 2017). A notable finding in this research was that social media served as an essential platform for resource-sharing, significantly contributing to the enhancement of community resilience. Social media enabled people to communicate their needs and provide resources to others, fostering a climate favorable to collective action. This discovery corroborates (Burke et al., 2010) assertion that social media amplifies social support by linking people to diverse support networks, including friends, family, and organizations. Social media offers a prompt and effective means for people to articulate their needs and address the needs of others (Christakis and Fowler, 2013). Participants noted the use of social media during the pandemic to request support or provide resources such as food, financial aid, or medical supplies. In my opinion, groups and Facebook have transformed into virtual platforms for individuals to disseminate urgent appeals, such as “Required: oxygen cylinders for a COVID patient,” which are then circulated across social networks. Participant 7 mentioned, he saw many requests for help in the messenger, was able to offer food and even donate some money to those who needed it. This discovery corresponds with (Goodyear et al., 2021) who observe that new media platforms might serve as a store of resources, facilitating access to essential services for people and families during catastrophes.

This habit of resource-sharing via social media platforms underscores its function in strengthening social connections and fostering a supportive community. Antoci et al. (2015) discovered that access to resources via social media adds to a feeling of belonging and social integration, both of which are crucial for developing resilience in times of disaster. Mano and Mano (2020) assert that social capital is cultivated via online social networks, whereby people experience more integration and support from their communities. The capacity to assist others, as shown via the acts of the participants, not only benefits others but also strengthens the helpers’ emotional resilience. Moreover, the community’s capacity for resource-sharing mitigated some of the stress associated with the economic pressures induced by the pandemic. During the crisis, Moitra et al. (2018) observed that social media served as a platform for expressing urgent needs and mobilizing help and resources, underscoring its larger importance in fostering community solidarity during crises. This research also found that social media cultivated an atmosphere conducive to prosocial behavior, considerably enhancing family and community resilience (Labrague, 2021). Participants observed that during the COVID-19 lockdown, social media channels enabled crowdsourcing initiatives to disseminate critical information and assistance, including the availability of hospital beds and oxygen cylinders. This kind of crowdsourcing exemplifies prosocial activity that may directly influence community resilience in times of crisis (Farrukh et al., 2023). During the peak of the pandemic, several research participants used imo, Facebook, and Messenger to disseminate information on the availability of crucial medical supplies.

One participant, Participant 5, he saw many posts about the lack of oxygen cylinders and hospital beds. People were sharing numbers, resources, and even offering to pick up supplies for others. According to Müller et al. (2016) this kind of crowdsourcing via social media platforms serves as a crucial resource for promoting community action during public health crises. Social media facilitated the dissemination of real-time information on accessible medical services, enabling families and communities to get essential assistance when traditional channels were inundated (Avery and Park, 2016; Qamar et al., 2021). Assert that social media platforms are essential for disseminating life-saving information during catastrophes. This research demonstrates that the dissemination of real-time information using social media platforms like Messenger and imo significantly mitigated the negative effects of the crisis on families, hence enhancing social resilience. Furthermore, social media served as a crucial venue for orchestrating community-oriented initiatives. Moitra et al. (2018) noted that during India’s COVID-19 crisis, social media acted as a platform for mobilizing volunteers who facilitated medical supply delivery and organized assistance efforts for individuals in need. These prosocial practices were essential in fostering community resilience, enabling communities to react effectively to systemic inadequacies in healthcare and resource allocation. Although social media offered considerable advantages in fostering resilience, it also posed obstacles. The overuse of social media, as noted by Gondwe et al. (2025), may result in digital exhaustion, with individuals expressing emotions of worry and tension. Ongoing exposure to distressing news, particularly about COVID-19 mortality, resulted in emotions of dread and anxiety. Participant 4 explained he had to limit my social media use because he was getting anxious from seeing too many sad stories and news updates. Furthermore, Ahmed et al. (2023) indicated that disinformation and rumors disseminated on social media might result in uncertainty, distrust, and potential injury. Participants indicated that managing contradictory information on social media on the virus, preventative strategies, and vaccine effectiveness increased their stress. As seen in the experiences shared by Participant 3 that he felt overwhelmed by the different opinions and news about COVID-19, and it made it difficult to know what to believe. Consequently, while social media provides significant assistance, it also poses the problem of navigating information overload and disinformation, which may erode emotional resilience.

## 6 Conclusion

The paper expands this theory by showing that digital resources have become central external assets that families turn to in order to ensure the functional efficiency during a crisis. It emphasizes that resilience is no longer an internalized mental quality but a digitally-mediated process since platforms such as Facebook and IMO play a very important role in their quality of life in terms of a vital process of protection in the family by means of isolation and financial pressure. The results confirm the multidimensionality of social support emotional, informational, and instrumental, in a digital environment. The research indicates that the new media contributes specifically to the meaning-making and sharing of resources and the difference between physical isolation and interdependence between people that is the core of the theory. Governments and regulators must devise policies that go beyond access to the internet to emphazise on important media literacy. This would enable the families to differentiate the good information and the false information that the research identified as a major source of anxiety. Regulators should consider the Digital Disparity observed in the results and be able to make family sustainability programmes accessible to the rural and low-income localities. The accessibility of these platforms to everyone is no longer a question of individual toughness and personal well-being.

It is necessary to have policies that facilitate the incorporation of official mental health hotlines and mental health counseling into the social media as they were found to be key lifelines to families.: There should be regulation of the digital intake. Although the families are encouraged to persist with the use of new media in connecting and gaining support, they need to strike a balance between this and offline activities to avoid the digital overload and stresses mentioned in the interviews. Developers of awareness programs should pay attention to designing virtual support communities where empathy and practical advice are the priorities rather than the dissemination of information. To facilitate optimal impact of digital content on family connectedness, the practitioners should make certain that the digital material is cultural and can be shared easily through widespread applications such as IMO and Messenger.

### 6.1 Limitations

Only 12 people were studied, limiting generalizability. A bigger, more diversified sample might reveal how new media affects family resilience across Jordan’s demographics and geographies (Pfefferbaum et al., 2017). Due to its concentration on Jordanian families, the results may not apply to other cultures. Due to cultural, sociological, and technical differences, new media may affect family sustainability differently in various nations (Kelle, 2006). Semi-structured interviews based on participants’ subjective accounts were used. This may lead to social desirability bias, as individuals exaggerate or reduce their usage of new media to meet expectations (Lim, 2025). New media usage is ubiquitous, but the research does not address the digital divide, especially in rural or underdeveloped regions where internet and digital device access is restricted. This impacts sample representativeness, particularly for families who may not fully participate with digital platforms owing to technical restrictions. New media may enhance resilience, but disinformation and misuse can harm it, according to the research. These concerns were not examined for their long-term implications on family resilience. The psychological and social effects of disinformation and excessive media consumption on families’ emotional and mental health require additional study.

### 6.2 Future research

Future research requires a larger, more diverse sample from Jordan’s many areas and comparisons with families from other countries. This would assist in understanding how cultural, socio-economic, and technical aspects affect family resilience via new media. Long-term research on how new media usage affects family sustainability and resilience may reveal how family life, mental health, and social networks change over time. Such research would examine family structure changes and how new media maintains resilience throughout prolonged crises. To understand how families respond to disasters, misinformation and family resilience must be studied. Research should examine digital disinformation and create media literacy measures to prevent its detrimental consequences. Understanding how families negotiate and manage disinformation might aid in establishing focused treatments to promote resilience during situations of uncertainty. Further research should study how digital and media literacy programs improve family resilience. It would be good to study how such programs influence families’ capacity to navigate new media responsibly, giving them the skills to detect dependable sources and manage their digital usage successfully. Research might address the digital gap and enable equal access to new media. We need studies on creative ways to increase technology access in underprivileged or rural locations to ensure all families can benefit from digital resilience-building methods. A network approach could provide deeper insights into the structural aspects of social support and offer a more detailed understanding of how support flows across different individuals and groups.

## Data Availability

The raw data supporting the conclusions of this article will be made available by the authors, without undue reservation.
